# Deep learning-based Alzheimer’s disease detection using magnetic resonance imaging and gene expression data

**DOI:** 10.1371/journal.pone.0330085

**Published:** 2025-08-18

**Authors:** Badar Almarri

**Affiliations:** Department of Computer Science, College of Computer Science and Information Technology, King Faisal University, Al Ahsa, Saudi Arabia; Jordan University of Science and Technology Faculty of Computer and Information Technology, JORDAN

## Abstract

Alzheimer’s disease (AD) poses significant challenges to healthcare systems across the globe. Early and accurate AD diagnosis is crucial for effective management and treatment. Recent advances in neuroimaging and genomics provide an opportunity for developing multi-modality-based AD diagnosis models using artificial intelligence (AI) techniques. However, the data complexities cause challenges in developing interpretable AI-based AD identification models. In this study, the author built a comprehensive AD diagnostic model using magnetic resonance imaging (MRI) and gene expression data. MobileNet V3 and EfficientNet B7 model was employed to extract AD features from gene expression data. The author introduced a hybrid TWIN-Performer-based feature extraction model to derive features from MRI. The attention-based feature fusion was used to fuse the crucial features. An ensemble learning-based classification model integrating CatBoost, XGBoost, and extremely randomized tree (ERT) was developed to identify cognitively normal (CN) and AD features. The proposed model was validated on diverse datasets. It achieved a superior performance on MRI and gene expression datasets. The area under the receiver operating characteristic (AUROC) scores were consistently above 0.85, indicating excellent model performance. The use of Shapley Additive exPlanations (SHAP) values improved the model’s interpretability, leading to earlier interventions and personalized treatment strategies.

## 1. Introduction

In the realm of neurodegenerative disorders, Alzheimer’s disease (AD) remains a significant challenge, affecting millions of individuals across the globe [1]. It progresses irreversibly and impairs individuals’ memory, reasoning abilities, and the ability to perform routine tasks [2]. The loss of neurons and synapses in the cerebral cortex is one of the notable characteristics of AD [1–3]. The identification of AD in the initial stages is crucial for effective treatment. The intricate interplay of genetic factors and brain structural changes introduces an additional layer of complexity to the AD disorder [4]. Due to the complexities of AD symptoms, advanced medical technologies face challenges in AD diagnosis [4]. Over the decade, an extensive range of approaches, including clinical assessments, neuroimaging, and genetic profiling, have been employed to diagnose AD.

Clinical assessment is the traditional and widely used approach for AD diagnosis [4]. Standardized tests, including the Mini-Mental State Examination, the Montreal Cognitive Assessment, and the AD Assessment Scale-Cognitive Subscale, play a significant role in various clinical processes in identifying AD [5]. The outcomes of these assessments are intended to evaluate impairments in a variety of cognitive abilities, notably memory, language, and problem-solving [6]. These tests distinguish severe AD from normal cognition [6]. However, they lack the sensitivity to identify AD in the early stages. Early AD symptoms, ranging from minor memory lapses to modest challenges in finding words, may indicate normal aging or stem from unrelated conditions. The examiner’s competence, cultural norms, and the patient’s education level may introduce a degree of subjectivity into these clinical and cognitive assessments [7]. Therefore, depending on clinical assessments may lead to an underdiagnosis or a misdiagnosis of early AD, leaving individuals without the opportunity to receive therapies to improve their quality of life.

Positron emission tomography can highlight protein deposits, including amyloid-beta and tau tangles, indicating AD [8]. However, it is expensive, less accessible, and may yield ambiguous findings, lacking in exhibiting clinically significant cognitive deficits. Magnetic resonance imaging (MRI) reveals brain structural and functional variations [8]. It supports researchers and medical professionals in assessing disease progression by detecting atrophy patterns, ventricular enlargement, and cortical thinning in the hippocampus, cortex, and other neurodegenerative locations [8]. These findings may assist in identifying early-stage AD and encourage early intervention. APOE gene variations and other AD-linked single nucleotide polymorphism (SNPs) may accelerate cognitive decline or alter disease progression in specific populations [9]. Incomplete risk assessment may result from relying on MRI, neglecting genetic and biological factors [9]. In addition, MRI-based analysis may misclassify or exclude early-stage patients without substantial structural alterations. Though more affordable than MRI or PET, computed tomography offers less information on the subtle brain structural changes essential to diagnose AD [10]. There is no single imaging modality that identifies AD individuals in the early stages. The mere existence of variations in imaging data cannot ensure AD manifestation due to its complex interplay of genetic, behavioral, and environmental factors [10]. Thus, neuroimaging may restrict tailored therapies based on each individual’s molecular composition.

The diverse nature of AD may reduce the generalization capability of automated tools for diagnosing AD [11]. With substantial amyloid plaques, some individuals may maintain their cognitive abilities for a long time. In contrast, individuals with no apparent imaging indicators can observe a rapid decline in their cognitive abilities [11]. These variations demonstrate the limitations of single-modality techniques and the demand for comprehensive models, capturing the multifaceted complexity of AD pathology.

A multi-modality technique, integrating multiple data sources and utilizing deep learning (DL) algorithms, is gaining attention as an alternative to provide accurate and rapid diagnoses [12]. Traditional models face challenges in handling enormous, complicated datasets with numerous variables. Implementing DL is essential to the process of utilizing multi-modal data. DL models, including convolutional neural networks (CNNs) along with additional architectural variations (e.g., recurrent networks, transformers), excel in finding patterns in high-dimensional data [13]. CNNs can recognize subtle variations associated with AD [14]. These models can handle tabular or vector-based inputs, such as genetic data. By analyzing multiple input streams, the models can recognize complex structural, biochemical, and clinical correlations.

Multi-modality-based DL models have multiple barriers. Data harmonization is the primary challenge [15]. MRI and genetics data differ in scale, format, and noise level. Normalizing, aligning, and preprocessing each modality requires a well-designed workflow. Due to the straightforwardness of enrolling cognitively normal individuals, AD research typically has class imbalances [16,17]. Without balanced training or augmentation, DL models may overfit the majority of classes [18]. Interpretability is crucial in DL-based AD detection [18]. The significant feature contributing to the outcomes is essential to improve the trustworthiness of the DL models. Recent developments, including Grad-CAM and attention mechanisms provide considerable transparency to the DL models’ outcomes [19].

Personalized risk management may be guided by genetic profiles, and imaging biomarkers can reveal crucial regions of the brain, requiring careful monitoring [20,21]. By leveraging these features, clinicians can provide personalized treatment, leading to successful intervention. Detecting AD in the initial stages can improve the individual’s quality of life. These factors motivate the author to develop a multi-modality DL model for early and accurate AD identification. The integration of MRI and genetic data into a comprehensive model can detect intricate AD patterns. In this study, the author builds a multi-modality-based AD detection model facilitating explainable outcomes.

The novel contributions of this research are listed as follows:

1. Hybrid feature extraction model for identifying crucial AD features.

A hybrid transformer model integrating Twin and Performer architectures is developed to extract features from MRI data. The synergy of MobileNet V3 and EfficientNet B7 is employed to extract meaningful features from gene expression data. These approaches are innovative in applying DL architectures for multi-modal data-based feature extraction.

1. A feature fusion technique using an attention mechanism.

The author introduces an attention mechanism combining features from MRI and genetic data. The attention mechanism prioritizes features through the computation of scores based on the importance of features.

1. Ensemble-Learning-based AD classification.

The core of the EL strategy lies in integrating the potential of XGBoost and CatBoost using extremely randomized tree (ERT). Unless traditional meta-learners, ERT introduces additional randomness to refine the final prediction. This approach captures a broader spectrum of AD patterns, enhancing model’s generalizability. In addition, this study facilitates the model’s interpretability by visualizing the features influencing the model’s decision.

The remaining part of this study is organized as follows:

Section 2 critically examines existing studies, highlighting key advancements in AD detection. It identifies knowledge gaps in current methodologies and justifies the need for the proposed AD detection approach. Section 3 presents the methodological approach, including data acquisition, feature extraction, and AD classification. The study findings are outlined in section 4. This section interprets data to evaluate the model’s effectiveness. Section 5 discusses the study’s implications and limitations. Lastly, section 6 synthesizes the study findings and suggests key areas for future research.

## 2. Literature review

The existing literature reflects the transition from clinical evaluations and single-modality assessments to integrated, technology-driven techniques. Although these methods were effective in diagnosing AD, they frequently struggled to identify subtle AD markers or precisely capture variation among individuals. Consequently, these procedures typically resulted in late-stage diagnoses, restricting the opportunities for successful AD intervention.

Chaitra and Shetty (2021) [22] used the DenseNet121 architecture to classify the MRI images. Jagadeeswari et al. (2022) [23] introduced a DL-based AD classification model. They used CNNs for the feature extraction and classification. Singh et al. (2023) [24] employed EfficientNet B5 for detecting AD. They applied the early stopping strategy to improve their model’s performance. Sait and Nagaraj (2024) [25] employed a hybrid CNN-vision transformer (ViTs) model for the feature extraction. They used a feature fusion technique to identify the intricate patterns in the MRI images. Similarly, Aghdam et al. (2024) [26], Pramanik et al. (2024) [27], and Khatri et al. (2024) [28] employed ViTs for AD risk classification using the MRI images.

Neuroimaging-based DL models exhibited potential in identifying AD, revealing structural brain alterations including cortical thinning and hippocampal atrophy. Even though the imaging markers significantly improved detection accuracy, there are notable gaps in AD diagnosis. The existing DL models face challenges in highlighting the early stages of disease progression. Neuroimaging-based models were unable to disclose the underlying genetic predispositions or molecular mechanisms.

Li et al. (2018) [29] proposed a model using gene expression data. They used LASSO regression for the feature selection. The majority voting approach was used to ensemble the outcomes of support vector machine (SVM), logistic regression (LR), and random forests (RF). Using genetic data, Lee and Lee (2020) [30] built an AD detection model. An auto-encoder architecture was used to identify the key features. The techniques, including LR, LASSO, RF, SVM, and deep neural networks were used to classify the extracted features. Wang et al. (2022) [31] utilized genetic and MRI data to detect AD. They extracted key features by integrating computer vision, natural language processing, and 3D-CNN to extract the key features. Abdelwahab et al. (2023) [32] proposed an AD detection model using genetic data. They used principal component analysis (PCA) and singular value decomposition to identify pertinent genes. Venugopalan et al. (2021) [33] proposed a multi-modal DL model for AD identification. Lew et al. (2023) [34] introduced a multi-modality-based AD detection approach. They employed bimodal Gaussian mixture model to process positron emission tomography (PET) scans. A CNN model was used to extract features from MRI scans. The individuals’ demographic data were utilized to support the model’s decisions. Feature fusion and classification techniques were used to generate the outcomes.

The integration of multi-omics data has been used to discover molecular markers associated with AD. The diagnosis performance can be improved by using sophisticated DL models integrating imaging characteristics with genetic data, the diagnostic performance can be improved. These methods enhance sensitivity in early detection. They offer opportunities for individualized risk assessment, exhibiting specific genetic variations and anatomical patterns of individuals. The findings of the literature review underscore the demand of effective approaches to address the limitations, including heterogeneity of datasets and lack of interpretability.

## 3. Materials and methods

**Fig 1:**
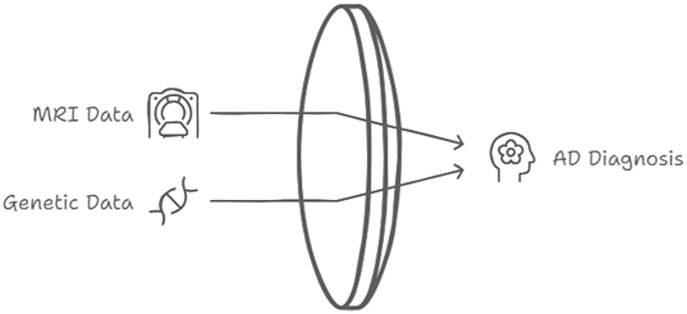
Integrating Multiple Data Sources.

The increased value of each modality to AD diagnoses supports combining MRI with gene expression data in the proposed model. MRI offers a rapid and non-invasive method for evaluating brain structures. On the other hand, gene expression enables a comprehensive investigation into the molecular processes related to AD. The synergy of these two modalities provides an extensive diagnostic tool. They uncover a wide range of AD pathology, from macroscopic anatomical abnormalities to microscopic molecular activity. The advanced DL techniques, including CNNs and SHAP, enable this data integration leading to an application that is scientifically robust and therapeutically meaningful. Fig 1 highlights the integration of MRI and genetic data, offering a powerful strategy for early detection of AD. MRI captures the changes in cortical thinning and hippocampal atrophy regions. Genetic data presents insights into individual predispositions. By leveraging these data, subtle brain abnormalities can be detected. Disease onset or progression may be expedited by correlating them with genetic markers.

**Fig 2:**
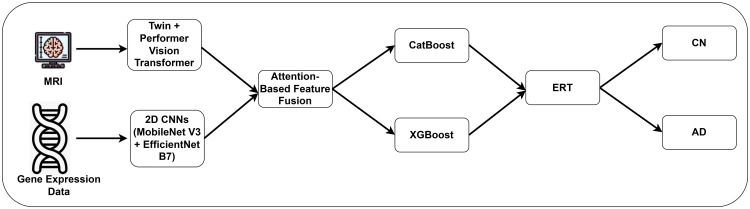
Proposed AD diagnosis approach.

Fig 2 illustrates the proposed methodology for AD diagnosis. It displays a comprehensive approach through the integration of MRI and gene expression data. TWIN-Performer transformers are selected for their ability to discern complex spatial relationships and contextual information. Additionally, 2D CNNs involving MobileNet V3 and EfficientNet B7 models, are used to analyze gene expression data. This approach enables a detailed and scalable analysis of genetic patterns, indicating predispositions or early signs of AD. By leveraging attention-mechanism-based feature fusion, the author captures the crucial features of cognitively normal (CN) and AD, maximizing information yields from each data type. To classify the features, an ensemble learning comprising CatBoost, XGBoost, and ERT models is employed. This integrated and multi-modal approach enhances the proposed model’s capability to identify nuanced patterns, addressing potential overfitting and generalization challenges.

### 3.1. Data acquisition

The authors utilize the AD Neuroimaging Initiative (ADNI) [35], the AddNeuroMed (ANM) [36] consortium, and the Open Access Series of Imaging Studies (OASIS) datasets to form a substantial foundation for AD detection. These datasets present diverse data, including MRI scans and extensive genetic probes. By leveraging diverse data, a comprehensive analysis can be conducted to investigate the factors contributing to AD.

ADNI [35] has been instrumental in AD research, comprising MRI scans, genetic data, and clinical assessments. The authors use ADNI 1, encompassing 550 individuals. This cohort provides a rich source of MRI scans obtained using 1.5 Tesla MRI equipment. In addition, the repository contains Ribonucleic Acid (RNA) samples of cognitively normal (CN) and AD individuals. The ANM consortium dataset [36] (ANM1 and ANM2) consists of gene expression data measured across numerous probes. It represents unique spots on a microarray used to simultaneously measure the gene expression levels. The comprehensive coverage of the genome enhances the likelihood of detecting subtle genetic variations associated with AD. Each individual contributes one diagnostic label: AD or CN. In the datasets, no samples are left unlabeled, and no subject appears with two different labels. Mild cognitive impairment or uncertain cases are excluded from the datasets. Each MRI samples and its matched genetic profile passed to the classifier as a single, fully specified example of either AD or CN, ensuring ground-truth supervision throughout the training, validation, and testing phases.

Table 1 outlines the dataset’s composition and multi-modal data availability. After applying rigorous quality control protocols, the ADNI dataset covers MRI and genetic data of 550 subjects, ANM1 provides fully matched MRI and genetic data of 341 subjects, and ANM2 contributes an additional 236 subjects, totaling 1127 individuals with comprehensive multi-modal information. OASIS [37] datasets include diverse neuroimaging data of subjects across different cognitive states. The authors utilize OASIS-1, focusing adults between 18 and 96. It includes MRI scans of 416 individuals. It offers longitudinal and cross-sectional data, supporting research studies on the AD progression and the states of the disorder at various stages.

**Table 1:** Datasets Composition and Multi-modal Data Availability.

Dataset	MRI	Genetic Modality	Overlap (subjects with MRI and genetic data)
ADNI	661	569	550
ANM1	458	369	341
ANM2	396	412	236
OASIS-1	416	Not Available	0

### 3.2. Data Preparation and Augmentation

Compared to the CNN architecture, ViT demands minimal MRI image preprocessing. Due to its robust attention mechanism, the need for complex data augmentation is minimized. However, the author employed data augmentation approaches to reduce the class imbalances. In addition, data normalization was used to adjust the intensity values across different scans to a standard scale. This approach counteracts variability between different MRI equipment. The images were divided into 16 × 16 pixels (patches), enabling the model to manage the computational load while providing necessary details. Based on the study [30], the author selected high-quality RNA samples. The median RNA expression value of ADNI 1 was 3,897. Using the intensity value, the background noise was reduced. The robust multi-array method was followed for data normalization. By leveraging the significance analysis of microarrays, the author extracted differentially expressed genes of CN and AD individuals. Substantial normalization and transformation approaches are followed to effectively prepare data for CNN processing. Z-Score normalization was employed to reduce variability caused by sequencing depth and RNA concentration differences. Subsequently, the Z-Scores are encoded using Min-Max scaling technique. This technique is straightforward and guarantees the existence of non-negative and bounded values. Finally, the scaled gene expression data are structured as a 2D matrix. Each row indicates a sample, and a column represents its corresponding gene.

Table 2 displays the class distribution of the datasets before and after data augmentation. Before data augmentation, datasets exhibit noticeable class imbalance. The author applies data augmentation techniques, including random rotation, random flipping, scale transformation, elastic deformation, intensity normalization, gene shuffling, and Z-score scaling with varying baselines, to address class imbalance and overcome model biases. The targeted data augmentation is employed specifically on the minority AD samples in each dataset. To overcome data leakage and maintain methodological rigor, the author divides the dataset based on the subject level rather than image or patch-level [38]. Specifically, the subjects are assigned to training (70%), validation (15%), and test (15%) subsets, stratifying by diagnostic label (AD or CN). The subject-level splitting ensures that the augmented images or patches derived from a single subject within the same partition (train set), preventing leakage and overlay optimistic performance estimation. No augmentation occurs on the validation and test sets, maintaining their integrity and unbiased measures of model performance [38]. In addition, the generated patches are assigned to the subsets based on their originating subjects. This strategy addresses concerns of patch-level leakage [38].

**Table 2:** Class Distribution Before and After Data Augmentation.

Dataset	Modality	Status	Total Samples	AD	CN	Class Ratio (CN:AD)	Augmentation Applied
ADNI	MRI + Genetic	Original	550	270	280	1.04:1	No
ADNI	MRI + Genetic	Post-Augmentation	1100	550	550	1:1	Yes
ANM1-ANM2	MRI + Genetic	Original	577	285	292	1.02:1	No
ANM1-ANM2	MRI + Genetic	Post-Augmentation	1040	520	520	1:1	Yes
OASIS-1	MRI only	Original	416	100	316	3.16:1	No

### 3.3. TWIN-Performer-based MRI Feature Extraction

The author integrated TWIN [39] and Performer [40] transformers for MRI feature extraction. The parallel integration leverages the unique capabilities of each transformer architecture, enhancing the interpretation of complex spatial and sequential patterns. Compared to the existing ViTs, the localized attention in TWIN and the kernalized approximation of Performer significantly reduce the complexities in the feature extraction process. Performer provides the global contextual insights, assisting in understanding the brain architecture variations. To reduce the computational costs, the author adds positional encodings to each patch in order to provide context about the absolute positions in the MRI scans. Eqn. 1 presents the feature extraction using the TWIN transformer.


$$F_{T}=TWIN\left(P_{i},\;A_{t}\right),\;i=1,2,\ldots .,\;N$$ (1)

where $F_{T}$ is the TWIN features, $P_{i}$ is the image patches, and $A_{t}$ is the attention mechanism.

TWIN transformer focuses on local window-based self-attention within patches, capturing fine-grained spatial relationships and localized features associated with CN and AD individuals.

Performer handles the patches in order to capture broader and global contextual relationships. It uses randomized feature maps, minimizing computational complexity. It enables the proposed model to learn dependencies across the MRI scans. Eqn. 2 outlines the process of the feature extraction using Performer.


$$F_{P}=Performer\left(P_{i},\;A_{t}\right),\;i=1,2,\ldots .,\;N$$ (2)

where $F_{P}$ is the Performer features, $P_{i}$ is the image patches, and $A_{t}$ is the attention mechanism.

A fully connected layer is used to concatenate the TWIN and Performer features. The combination of TWIN and Performer captures the intricate and larger structural contexts critical for accurate diagnosis. Eqn. 3 highlights the feature fusion strategy.


$$\begin{array}{c} F_{MRI}=\sum_{i=1}^{N}F_{T_{i}}+F_{P_{i}} \end{array}$$ (3)

$F_{MRI}$ is the concatenated features, + is the concatenation operation, $F_{T_{i}}$ and $F_{P_{i}}\;$are the TWIN and Performer features, respectively.

### 3.4. MobileNet V3-EfficientNet B7-based gene expression data feature extraction

In order to extract complex biological features, the author employs the sequential integration of MobileNet V3 [41] and EfficientNet B7 [42]. MobileNet V3 processes the 2D matrix using the lightweight depthwise separable convolutions. It extracts salient features with minimal loss of crucial information. The initial layers capture basic patterns in the gene expression, identifying primary relationships and clusters among gene activities. Eqn. 4. Provides the feature map generation process.


$$f_{M}=\;\sigma \left(BatchNorm\left(DepthwiseConv(X)\right)\right)$$ (4)

where $f_{M}$ is the feature map, σ is the non-linear activation function, and X is the 2D matrix.

EfficientNet B7 handles the feature maps to refine the feature maps. It uses a compound coefficient to scale network width, depth, and resolution. By adjusting varying scales of features, the feature representations are enhanced. The subsequent layers of EfficientNet B7 capture complex CN and AD patterns. Eqn. 5 shows the computational model for refining features.


$$E_{F_{m}}=EfficientNet\;B7\left(f_{M}\right)$$ (5)

where $E_{F_{m}}$ is the feature maps of the EfficientNet B7 and $f_{M}$ is the MobileNet V3’s feature maps.

A custom regularization and adaptation phase is used to fine-tune the final feature maps. This phase uses layer-wise fine-tuning with attention mechanism emphasizing regions of high variability, indicating significant biological events. The sequential integration approach guarantees that each stage of the feature extraction adds value, advancing the capability of genomic analysis. Eqn. 6 presents the features extracted through the MobileNet V3-Efficient B7 models.


$$F_{Gene}=Dense\left(Attention\left(E_{F_{m}}\right)\right)$$ (6)

where F is the MobileNet V3-EfficientNet B7 features, and $E_{F_{m}}$ is the EfficientNet B7’s feature maps.

### 3.5. Attention-mechanism-based fusion

An attention-mechanism-based feature fusion strategy is employed to combine the most relevant features extracted from the TWIN-Performer and MobileNet V3-EfficientNet B7 models. Eqn. 7 presents the concatenation of the features.


$$F_{C}=\;\left(F_{MRI}\&F_{Gene}\right)$$ (7)

where $F_{C}$ is the concatenated features, $F_{MRI}$ is the MRI features, $F_{Gene}$ is the gene expression features, and "&" denotes the concatenation along the column, generating single extended feature set.

Subsequently, an attention mechanism is used to refine features. Based on the concatenated features, the attention weights are computed as shown in Eqn. 8.


$$\alpha =Softmax\left(W.F_{C}+b\right)$$ (8)

where W is the weight matrix, α is the attention weight, b is a bias vector, and $F_{C}$ is the concatenated features.

The Softmax function is used to ensure that the attention weights sum to one. The final fused feature is computed as a weighted sum of the concatenated features. Eqn. 9 shows the mathematical expression of fused feature vector.


$$F_{fused}=F_{C}.\alpha^{T}$$ (9)

where $F_{fused}$ is the final feature vector, $\alpha^{T}$ allows for the multiplication along the appropriate dimension, and$\;F_{C}$ is the concatenated features.

Moreover, the author applied principal component analysis to reduce the dimension of the fused feature vectors. PCA supports in mitigating the risk of overfitting. In addition, it enhances the model’s classification accuracy by capturing the significant variance and patterns.

### 3.6. EL-based AD classification

CatBoost [43] and XGBoost [44] models are trained using the fused feature vectors. These models can capture different aspects of the fused features due to differences in the depth of trees and learning rates. They generate outcomes using their unique approaches. Eqns. 10 and 11 show the individual predictions of CatBoost and XGBoost models.


$$P_{CatBoost}=CatBoost\left(F_{fused}\right)$$ (10)

$$P_{XGBoost}=XGBoost\left(F_{fused}\right)$$ (11)

where $P_{CatBoost}$ and $P_{XGBoost}$ are the predictions of CatBoost and XGBoost, accordingly and $F_{fused}$ is the final feature vector.

Using ERT [45] to ensemble the intermediate predictions reduce variance and bias from individual model predictions. ERT minimizes overfitting by averaging over a large number of de-correlated trees. It introduces additional randomness in the tree construction that contributes to a stable aggregation, generating consistent and reliable predictions. A modality masking mechanism is introduced into the proposed AD classification pipeline, addressing the absence of genetic data. To fine-tune the ERT, the author employs Bayesian Optimization and HyperBand (BOHB) algorithm. BOHB optimizes ERT by balancing exploration (Bayesian Optimization) and exploitation (HyperBand). Eqn. 12 presents the computation of the final decision with the SHAP values.


$$P_{final},\;SHAP=SHAP\_function\left(ERT\left[P_{CatBoost},P_{XGBoost}\right]\right)$$ (12)

where $P_{final}$ is the final prediction, SHAP is the value influencing the decision, and $P_{CatBoost}\;and\;P_{XGBoost}$ are the intermediate decisions.

## 4. Results

To implement the proposed AD detection, the author uses Windows 10 Pro, Intel i7 – 12700K, and NVIDIA GeForce RTX 3080. In addition, 32 GB DDR4 and 1 TB NVMe SSD are used to manage large datasets and store extensive model weights. The author employs Python 3.8, TensorFlow 2.4, Pytorch 1.7., CUDA 11.0., CUDNN 8.0., and SHAP libraries to build the model. The datasets are divided into train set (70%), validation set (15%), and test set (15%). This experimental setup offers a powerful and flexible environment for developing an AD detection model using the cutting-edge DL models. The performance metrics include accuracy, precision, recall, F1-Score, specificity, and the area under the operating characteristic (AUROC). AUROC offers robust discrimination measurement when class proportions are approximately equal. It is entirely justified and reliable as an overall performance metric. These metrics are used to guarantee a robust evaluation by addressing different aspects of model’s performance, providing a transparent and nuanced assessment of model performance.

The author trains the model separately on the ADNI and the combined ANM1-ANM2 datasets. The ADNI and ANM projects differ significantly in the MRI acquisition, scanner manufacturers, image resolution, and genotyping platforms. The dedicated training on the individual datasets enables the model to learn dataset-specific patterns without inadvertently modeling acquisition-related biases as disease signals. By training and validating across datasets, the model’s generalization is improved. This strategy provides more substantial evidence for the biological validity and clinical robustness of the discovered imaging and genetic features. Genotyping arrays and quality-control procedures differ across ADNI and ANM datasets. Thus, separate training guarantees better handling of genomic data differences. Although training is conducted separately, the findings can be compared and analyzed. This training strategy strengthens the validity of the conclusions drawn from each dataset and their generalizability to clinical environments. Table 3 outlines the configuration details for implementing the proposed AD detection model. It presents the key parameters of the feature extraction and fusion, and EL-based classification models. Each entry, including the number of layers, dropout rates, and other critical hyperparameters, guarantees transparency and reproducibility of the proposed models.

**Table 3:** Computational Strategies.

Model	Description	Values
TWIN-Performer	Convolution layers	3
	Transformer layers	5
	Dropout	0.2
	Number of attention heads	12
	Feature dimensions	512
MobileNet V3-EfficientNet B7	MobileNet V3 layers	Configured for minimal depth
	EfficientNet B7 scaling	Default scaling parameters
	Dropout	0.2
	Activation functions	Swish in EfficientNet B7
Feature Fusion	Multilayer perceptron with attention	2
	Dropout	0.15
	Attention scaling	Softmax across features
CatBoost	Depth	8
	Learning rate	0.03
	Iterations	1500
XGBoost	Maximum depth	7
	Learning rate	0.2
	Number of estimators	300
ERT	Number of trees	150
	Regularization	L1/ L2

**Fig 3:**
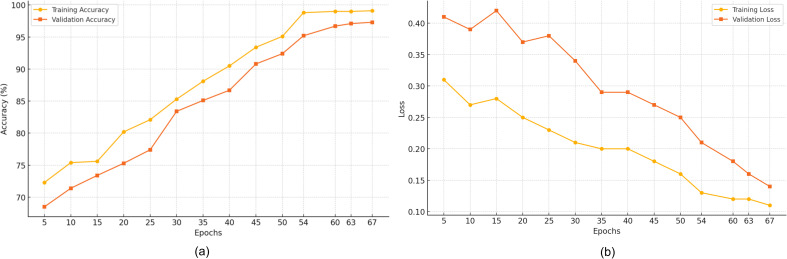
Findings of Training and Validation Phases Using ADNI Dataset (a) Accuracy and (b) Loss.

Fig 3 illustrates the outcomes of the training and validation for the proposed model trained on the ADNI dataset (training and validation sets). The exponential increase in the training accuracy suggests that the model is effectively learning from the training data. The close tracking between of validation accuracy and loss with training counterparts indicates the generalization ability. The slight discrepancies between training and validation highlight the areas for model refinement, enhancing the model’s stability on unseen data.

**Fig 4:**
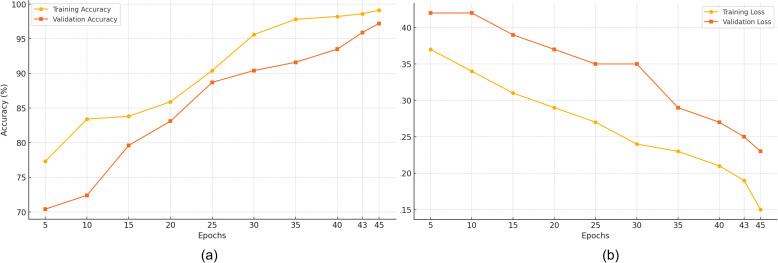
Findings of Training and Validation Phases Using ANM1-ANM2 Dataset (a) Accuracy and (b) Loss.

Fig 4 outlines the performance dynamics of the proposed model across various epochs using ANM1-ANM2 dataset (train and validation sets). It reveals a possible trajectory in the model’s accuracy at different epochs. The training and validation loss descents are smooth and consistent. The early stopping strategy and regularization technique supported the model to prevent overfitting. PCA produces an effective set of orthogonal components that captures the influential features associated with AD and CN patterns. This dimensionality reduction strategy improves the performance of the ensemble classifier, demonstrating faster convergence and consistent performance compared to the models trained on the entire feature set. PCA-based proposed classifier reports higher accuracy and F1-score. The PCA’s inherent transparency facilitates the interpretation of individual principal components in terms of the underlying biological features. Through the ensemble classifier, the proposed AD classification pipeline classifies the features using principal components, enabling the detection of complex and non-linear relationships across modalities.

Table 4 demonstrates the robustness and reliability of the proposed AD detection model in diagnosing AD using MRI. The slight variations in the model’s performance between ADNI and OASIS (20%) can be attributed to differences in image quality. The high scores underscore the model’s potential in identifying CN and AD individuals. The suggested feature extraction and fusion techniques enhanced the model’s classification accuracy.

**Table 4:** Performance Evaluation -MRI (Test set).

Classes	Accuracy	Precision	Recall	F1-Score	Specificity
ADNI					
CN	98.7	97.8	97.7	97.7	97.3
AD	98.9	98.2	98.6	98.4	98.6
Average	98.8	98.0	98.1	98.0	97.9
OASIS					
CN	98.5	97.1	98.1	97.6	98.1
AD	98.8	96.9	97.4	97.1	96.4
Average	98.6	97.0	97.7	97.3	97.2

The outcomes demonstrated in Table 5 underscore the capabilities of suggested feature fusion and classification approaches. The model obtains a high average accuracy of 91.9% on the ADNI dataset. The findings indicate the model’s strength in identifying true positive cases. The average accuracy of 90.0% on the ANM1-ANM2 dataset highlights better generalization across different datasets.

**Table 5:** Performance Evaluation – Gene expression data (Test set).

Classes	Accuracy	Precision	Recall	F1-Score	Specificity
ADNI					
CN	91.5	90.2	89.3	89.8	90.2
AD	92.3	89.7	88.8	89.2	91.3
Average	91.9	89.9	89.0	89.5	90.7
ANM1-ANM2					
CN	90.5	89.1	87.9	88.5	88.9
AD	89.5	88.5	88.1	88.3	90.1
Average	90.0	88.8	88.0	88.4	89.5

Table 6 displays the performance of the proposed model and various pre-trained architectures on the ADNI and OASIS datasets. The proposed model shows superior performance compared to other models. It achieved excellent generalization performance on the OASIS dataset. The hybrid transformer architectures enable a holistic analysis of AD indicators. The suggested attention-based feature fusion contributes to the classification decisions, enhancing accuracy and specificity.

**Table 6:** Comparative Analysis (Pre-trained Models) – MRI (Test set).

Classes	Accuracy	Precision	Recall	F1-Score	Specificity	Peak GPU RAM (in GB)	Inference Latency (GPU, single Scan) (in ms)
Proposed Model (ADNI)	98.8	98.0	98.1	98.0	97.9	11.8	32 ± 3
Proposed Model (OASIS)	98.6	97.0	97.7	97.3	97.2	11.8	38 ± 4
TWIN	95.6	93.2	92.4	92.8	91.9	12.9	45 ± 8
SWIN	96.7	92.8	90.8	91.8	91.5	13.5	41 ± 4
LeViT	97.1	91.9	91.1	91.5	90.8	12.2	39 ± 5
Linformer	95.0	90.8	89.8	90.3	90.7	10.4	51 ± 3
Performer	96.8	91.5	90.4	90.9	91.1	11.2	55 ± 3

Table 7 reveals the findings of a comprehensive comparative analysis using gene expression data. The use of MobileNet V3-EfficientNet B7 handles the gene expression data effectively, enhancing the model’s specificity. The innovative feature fusion strategy provides crucial features that contribute to CN and AD. The model outcomes underscore the potential of advanced fine-tuning an optimization strategy. The extensive pre-processing and feature extraction reduced the complexities of gene expression data.

**Table 7:** Comparative Analysis (Pre-trained Models) – Gene expression data (Test set).

Classes	Accuracy	Precision	Recall	F1-Score	Specificity	Peak GPU RAM (in GB)	Inference Latency (GPU, single Scan) (in ms)
Proposed Model (ADNI)	91.9	89.9	89.0	89.5	90.7	11.8	39 ± 4
Proposed Model (ANM1-ANM2)	90.0	88.8	88.0	88.4	89.5	11.8	41 ± 5
MobileNet V3	87.7	84.6	83.8	84.2	85.5	9.5	61 ± 4
EfficientNet B7	86.9	85.6	84.9	85.2	83.8	15.4	71 ± 8
DenseNet 264	83.7	81.5	82.6	82.0	85.9	18.6	54 ± 4
EfficientNet V2	87.9	82.9	82.5	82.7	84.5	17.4	46 ± 7
RegNetX	86.5	84.1	83.1	83.6	82.9	19.3	63 ± 4

The fusion strategies, including cross-attention and gated multi-modal units, require exclusive computational overhead. Cross-attention demands a dedicated attention map for each direction (MRI to genome and genome to MRI), increasing the training time by approximately 50% without yielding a significant classification improvement. Gated multi-modal units integrate modalities into a single scalar weight. It lacks training exposure to partial inputs. In contrast, the self-attention mechanism enables the model to identify the effective combination of anatomical and molecular features associated with AD. Moreover, it is inherently tolerant to missing data, allowing the architecture to be deployed on single-modality cohorts without necessitating additional architecture modifications.

**Fig 5:**
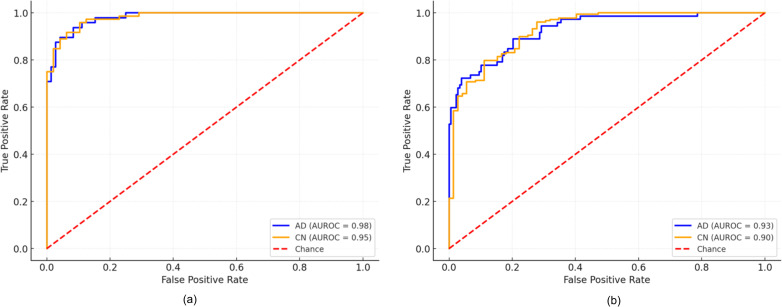
AUROC (a) ADNI – MRI (b) OASIS – MRI.

Fig 5 demonstrates the AUROC, highlighting the model’s advanced capability to utilize MRI for the reliable AD diagnosis. Although the model generates one probability per scan, clinical practice demands two distinct error types: misdiagnosing a normal subject as AD and missing an AD case. In order to highlight these asymmetric consequences, the author applies the dual reporting strategy, demonstrating the importance of the proposed model in protecting healthy individuals from overdiagnosis and reliably capturing actual AD cases. The proposed feature extraction using TWIN + Performer and MobileNet V3 + EfficientNet B7 offers the best combination of biological relevance, parameter efficiency, and empirical performance. The two-layer multi-layer perceptron (on the MRI images) lacks the ability to exploit the biologically meaningful spatial locality embedded in the two-dimensional grid, exhibiting an average AUROC of 0.85, lower than the proposed model. Similarly, on the MRI images, the one-dimensional CNN’s sequential ordering conflates distant chromosomal regions, achieving moderate performance with an average AUROC of 0.88. In contrast, the AUROC for CN and AD reaches 0.90 and 0.93 on the OASIS dataset, indicating the model potential in differentiating CN and AD in unseen data. The findings highlight the model’s scalability and reliability, enhancing personalized medicine approaches. The strategic use of cutting-edge techniques improves the model’s capability for the integration into clinical workflows. The proposed feature extraction achieves a remarkable outcome while maintaining better precision-recall scores. The superior trade-off between performance and computational resources indicates the significance of the proposed feature extraction approach, justifying the use of TWIN+Performer and MobileNet V3 + EfficientNet B7.

**Fig 6:**
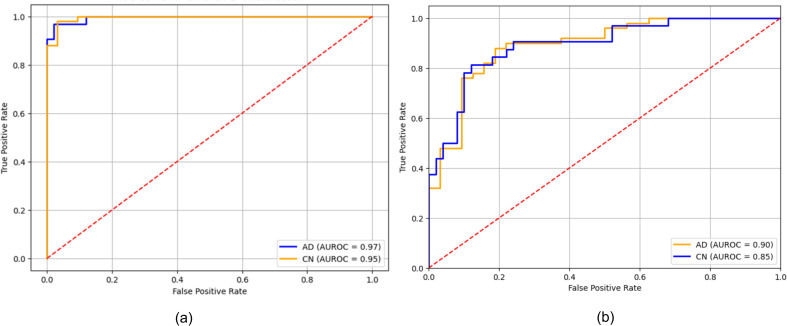
AUROC (a) ADNI (b) ANM1-ANM2.

Fig 6 illustrates the performance of the proposed model on the ADNI and ANM1-ANM2 datasets. The high score of CN on the ADNI indicates the robust performance in correctly identifying CN individuals, preventing false positive AD diagnoses. However, in ANM1-ANM2 dataset, the AUROC of CN is notably lower, suggesting variability in the model’s effectiveness in identifying individuals in different data settings. The lower score suggests room for further improvement in model tuning on additional datasets.

Table 8 highlights the application of SHAP values in analyzing the significance of specific features. It presents the predictive importance and clinical relevance of different biomarkers. A mean SHAP value of 0.57 substantially impacts AD risk prediction. Likewise, the negative SHAP value of −0.30 indicates the importance of Gene B in AD diagnosis. The gene expression plays a crucial role in decreasing AD risk. The integration of SHAP values provides a transparent view of features influencing the AD prediction. In order to perform feature importance analysis, the author employs TreeSHAP. ERT is an ensemble of decision trees. TreeSHAP enhances the ERT’s performance compared to the model-agnostic KernelSHAP. KernelSHAP relies on thousands of perturb-and-re-evaluate samples, requiring longer runtimes and introducing Monte Carlo noise into the estimates. In contrast, TreeSHAP leverages the hierarchical structure of decision trees, maintaining consistency and improving feature importance estimates. Using TreeSHAP, the local feature attributions are generated, enabling clinicians to interpret each prediction in terms of the specific MRI regions and gene expression markers related to the model’s decision. The single-output architecture overcomes conflicting class labels across modalities, maintaining interpretability using TreeSHAP. It improves discrimination ability compared to single modality models. Moreover, TreeSHAP computation reduces the runtime costs and instability associated with KernelSHAP.

**Table 8:** Sample Outcomes with SHAP Values.

Feature type	Feature ID	Mean SHAP value	Impact on prediction	Clinical relevance
MRI	Hippocampal volume	0.57	Increased risk	Decreased volume related to AD progression
MRI	Cortical thickness	0.32	Increased risk	Regions thinning associated with AD
Gene	Gene B	−0.30	Decreased risk	Protective; associated with cognitive resilience
Gene	Gene X	0.05	Increased risk	Minor role in synaptic function
MRI	Ventricular volume	0.28	Increased risk	Enlargement correlates with AD severity

Table 9 outlines a detailed comparative analysis of various AD models. It highlights the superiority of the proposed model on the test sets (ADNI and ANM1-ANM2) and OASIS dataset (20%) in terms of performance metrics and methodological advancements. The proposed model surpassed the recent models by achieving a generalization accuracy of 98.6% on the MRI datasets. Similarly, it outperformed AD models based on gene expression data. Sait and Nagaraj (2024) and Abdelwahab (2023) achieved superior performance on MRI and gene expression data. However, these models were based on single modality data, reducing their generalization capabilities.

**Table 9:** Comparative Analysis (Existing Studies).

Methods	Data Source	Feature Selection Approach	Classification Approach	Interpretability	Multi-Modality	Performance
Proposed Model	MRI	TWIN-Performer model	Ensemble Learning	Partial	Yes	Accuracy: 98.8% Precision: 98.0% Recall: 98.1% F1-Score: 98.0% Specificity: 97.9%
Proposed Model	MRI	TWIN-Performer model	Ensemble Learning	Partial	Yes	Accuracy: 98.6% Precision: 97.0% Recall: 97.7% F1-Score: 97.3% Specificity: 97.2%
Proposed Model	MRI + Gene Expression Data	MobileNet V3-EfficientNet B7	Ensemble Learning	Partial	Yes	Accuracy: 91.9% Precision: 89.9% Recall: 89.0% F1-Score: 89.5% Specificity: 90.7%
Proposed Model	MRI + Gene Expression Data	MobileNet V3-EfficientNet B7	Ensemble Learning	Partial	Yes	Accuracy: 90.0% Precision: 88.8% Recall: 88.0% F1-Score: 88.4% Specificity: 89.5%
Lee and Lee (2020) [30]	Gene Expression Data	LASSO regression	RF, SVM, and deep neural network	No	No	ADNI – AUROC: 0.657 ANM1 -AUROC: 0.874 ANM2- AUROC: 0.804
Abdelwahab et al. (2023) [32]	Gene Expression Data	PCA and singular value decomposition	singular value decomposition	No	No	Accuracy: 96.0% Loss: 0.35
Venugopalan et al. (2021) [33]	Electronic Health records, SNPs, and Imaging	Autoencoders and 3D-CNNs	3D-CNNs (fully Connected layer)	Yes	Yes	Accuracy: 84% Precision: 83% Recall: 83% F1-Score: 83%
Wang et al. (2022) [31]	Genetic and MRI	Computer vision and 3D-CNNs	3D-CNNs (fully Connected layer)	No	Yes	Accuracy: 83.78% AUROC: 0.924
Lew et al. (2023) [34]	PET, MRI, and clinical data	2D CNNs	2D CNNs (fully connected layer)	No	Yes	Amyloid – AUROC: 0.79 Tau-AUROC: 0.73 Neurodegeneration – AUROC: 0.86
Sait and Nagaraj (2024) [25]	MRI	ViTs and CNNs	CatBoost model	Yes	No	Accuracy: 98.8% Precision: 97.9% Recall: 98.1% F1-Score: 98.0% Specificity: 97.5%
Aghdam et al. (2024) [26]	MRI	ViTs and CNNs	ViTs (fully Connected layer)	No	No	Accuracy: 97.3% Precision: 96.1% Recall: 96.3% F1-Score: 96.2% Specificity: 90.1%
Pramanik et al. (2024) [27]	MRI	ViTs	ViTs (fully Connected layer)	No	No	Accuracy: 97.3% Precision: 97.4% Recall: 97.3% F1-Score: 97.3% Specificity: 95.1%
Khatri et al. (2024) [28]	MRI	ViTs	ViTs (fully Connected layer)	No	No	Accuracy: 91.3% Precision: 90.5% Recall: 90.7% F1-Score: 90.6% Specificity: 91.6%

## 5. Discussions

In this study, a novel AD detection model is presented. This model utilizes a cutting-edge CNNs and ViTs architectures for extracting features and an ensemble learning approach for feature classification. In order to focus on crucial biomarkers, the use of an attention-based fusion strategy is utilized, presenting highly discriminative features to CATBOOST and XGBoost models to generate intermediate outcomes. These outputs are refined through the ERT meta-learner model. The author generalizes the model on the OASIS and the ANM1-ANM2 datasets. The model achieves an impressive classification accuracy of 98.6% and specificity of 97.2 on the OASIS dataset and accuracy of 90.0% and specificity of 89.5% on the ANM1-ANM2 dataset. Moreover, the model’s outstanding discriminative capability between AD and CN cases was shown by its consistently high AUROC scores. Clinicians and researchers can gain crucial insights into the disease’s biomarkers and contributing factors to the application of SHAP values for interpretability. The complex etiology of AD is better understood with this multimodal approach, providing a broader perspective of the disorder compared to the existing methods.

Integrating structural MRI data with molecular biomarkers associated with AD enable a comprehensive investigation of the relationship between genetic predispositions and the brain’s phenotypic expression. For instance, MRI characteristics such as hippocampus volume and cortical thickness provide spatial and morphological insights. These insights with gene expression data can reveal biological processes at the cellular level, enriching the understanding of the disease’s progression. The comprehensive view of individual’s data allows healthcare practitioners to tailor treatment plans and targeted interventions. The synergy of neuroimaging and gene expression data-based analysis supports in identifying subpopulations that may respond differently to therapies. It fosters earlier interventions and significantly improves the understanding of AD mechanisms. While enhancing diagnosis and therapy, the proposed model may improve the performance of neurodegenerative disease prevention approaches. Individuals at high risk of developing AD may be identified before the manifestation of clinical symptoms, allowing clinicians to recommend preventive medications or modifications to lifestyles based on risk profiles. Dietary modifications, cognitive therapy, and exercise may be recommended to address genetic vulnerabilities.

In spite of its remarkable outcomes, the model encounters challenges such as data heterogeneity across multiple datasets (ANM, OASIS, and ADNI) and inherent variability in gene expression measurements. Due to the data complexity, the extensive data normalization procedures are required to reduce the potential biases. The computational complexity of advanced neural networks and model interpretation challenges demand future enhancements. The proposed AD classification architecture precludes the inclusion of rare-coding variants contributing to AD risk. The incorporation of whole-genome sequencing enables the model to capture low-frequency genetic variants. The use of molecular features, including brain-tissue DNA-methlyation signatures and histone modification quantitative trait loci can enhance the performance of the multi-modality models. Larger cohorts with MRI and molecular profiles can support robust and generalizable multi-modality models. Developing a reliable framework to address the technical challenges in handling ultra high dimensionality can lead to accurate early AD diagnosis and mechanistic insights into disease progression.

Furthermore, substantial research studies are essential to improve the data’s interpretability and clinical value, leading to an explainable AD diagnosis. Using finer-grained time-series data, future studies may investigate disease trajectories and predict AD in the early stages. Integrating cerebrospinal fluid biomarkers or rich clinical information may enhance the model’s contextual sensitivity, facilitating patient categorization and treatment. Advanced explainability techniques may simplify decision processes and boost clinician confidence and acceptance. The model’s generalizability and the likelihood of genetic or demographic differences may be addressed by testing it across larger more diverse datasets. Thus, extending the proposed study can allow healthcare professionals to diagnose AD in the initial stages.

## 6. Conclusions

This study presents significant advancements in AD diagnosis. It employed a synergistically combined cutting-edge approach to handle the complexities in MRI and gene expression data, enhancing the AD classification accuracy. The TWIN-Performer and MobileNet V3-EfficientNet B7 models enabled the proposed model to visualize brain morphology and gene expressions. The suggested feature fusion with PCA identified informative features. The application of BOHB fine-tuned the ensemble learning-based classification. The experimental results demonstrated exemplary performance of the proposed model, showcasing its discriminative ability in identifying CN and AD cases. The SHAP values offer clinicians and researchers with valuable insights into the disease biomarkers. The enhanced diagnostic accuracy can significantly improve patient outcomes through timely and targeted interventions. The proposed methodology provides a platform for exploring other complex diseases. It enhances the potential for discovering novel therapeutic targets. Future research should validate and refine the proposed model across diverse populations. In addition, real-world clinical trials are essential to assess the practical viability of the model in a clinical setting.
